# Histocompatibility and Reproduction: Lessons from the Anglerfish

**DOI:** 10.3390/life12010113

**Published:** 2022-01-13

**Authors:** Noah Isakov

**Affiliations:** The Shraga Segal Department of Microbiology, Immunology and Genetics, Faculty of Health Sciences, Ben Gurion University of the Negev, P.O. Box 653, Beer Sheva 84105, Israel; noah@bgu.ac.il; Tel.: +972-8-6477-267

**Keywords:** anglerfishes, sexual parasitism, histoincompatibility, allograft rejection, immune tolerance, adaptive immune response

## Abstract

Reproduction in certain deep-sea anglerfishes involves the permanent attachment of dwarf males to much larger females and fusion of their tissues leading to the establishment of a shared circulatory system. This unusual phenomenon of sexual parasitism enables anglerfishes to maximize reproductive success in the vast and deep oceans, where females and males otherwise rarely meet. An even more surprising phenomenon relates to the observation that joining of genetically disparate male and female anglerfishes does not evoke a strong anti-graft immune rejection response, which occurs in vertebrates following allogeneic parabiosis. Recent studies demonstrated that the evolutionary processes that led to the unique mating strategy of anglerfishes coevolved with genetic changes that resulted in loss of functional genes encoding critical components of the adaptive immune system. These genetic alterations enabled anglerfishes to tolerate the histoincompatible tissue antigens of their mate and prevent the occurrence of reciprocal graft rejection responses. While the exact mechanisms by which anglerfishes defend themselves against pathogens have not yet been deciphered, it is speculated that during evolution, anglerfishes adopted new immune strategies that compensate for the loss of B and T lymphocyte functions and enable them to resist infection by pathogens.

## 1. Introduction

The morphology and the feeding strategies of the eerie-looking deep-sea anglerfishes (*Ceratioidei*) have been shaped during millions of years of evolution in the eternal darkness of the deep oceans [1,2]. Most are characterized by having a globular body with a huge head and enormous crescent-shaped mouth that is filled with long, fang-like teeth. Their retractable jaw, lack of ribs, and overall pliable bodies enable them to feed on prey that is almost twice their own size [3]. In addition, they have perfected their feeding strategy by developing a specialized luminescent lure organ that enables them to attract prey in the hostile environment of the dark ocean [4]. This fishing rod-like apparatus, termed illicium (from the Latin word “*Illicio*”, which means “to entice”), is derived from a modified dorsal-fin spine which extends from the dorsal region of their head and is tipped with a sphere-shaped luminescent organ, called *ēsca* (which means “bait” in Latin) [4,5].

Most anglerfishes are unable to produce light on their own. Instead, they acquire the ability to glow from symbiotic bio-luminescent bacteria, predominantly from the *Enterovibrio* family (such as *E. escacola* and *E. luxaltus*) [6,7,8], which take up residence in the *ēsca*. Each species of anglerfish pairs with a unique species of luminescent bacteria [9], and the light, which is amplified by crystals and reflectors, is emitted through a transparent tissue. Anglerfishes can wiggle their illicium while keeping their body motionless, to better mimic a living bait, and reveal their shiny lure on demand. Thus, by pulsing the light and moving the lure back and forth, the anglerfish attracts crustaceans and fishes and snatches them before they have a chance to swallow the *ēsca*. The bioluminescent lure also functions as a male-attracting device, which increases the probability of finding mates in the vast oceanic space [10,11].

The length of the anglerfishes varies from 2 to 18 cm, with a few types growing up to 100 cm. The extreme size variations are due to the sexual dimorphism in which tiny males are up to an order of magnitude smaller than their females [2].

**Figure 1:**
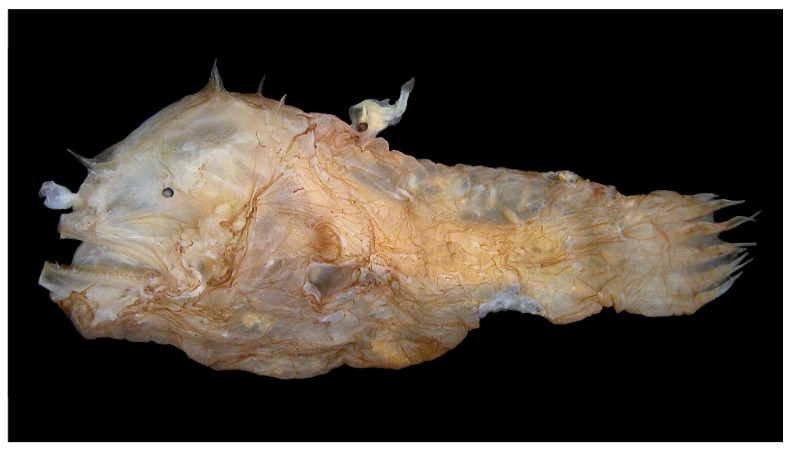
A female deep-sea anglerfish (*Photocorynus spiniceps*) with a dwarf parasitic male fused to her back. The male’s nutrients are provided by the female via the joined blood systems and, in turn, the male provides sperm on demand. This phenomenon of parabiosis persists despite a genetic disparity between the male and the female that shows no signs of an immune response against the histoincompatible antigens. Photo credit to Theodore W. Pietsch, University of Washington.

An even more unusual and distinctive characteristic of many anglerfish species is related to their mode of reproduction. The free-swimming tiny male anglerfish can sniff the female’s waterborne pheromones and latch itself onto the female with its sharp teeth. While in some species, male attachment to females occurs temporarily and does not involve fusion of tissues, in other species, the male releases enzymes that dissolve the female’s tissue around his mouth, leading to anatomical joining of the male and female tissues [1]—a naturally occurring form of parabiosis [12] (see Figure 1). Thereafter, the male loses its eyes, fins, and internal organs, except for its testes. Its blood system fuses with that of the female host, and from that point on it receives all its nutrients via the joined circulatory system.

The parasitic anglerfish male remains attached to the female throughout her lifetime, stays reproductively functional, and participates in multiple spawnings. Furthermore, to increase the fertilization probability, females of some anglerfish species form simultaneous parabiosis with multiple males. A report by Saruwatari [13] revealed eight males that were attached to a single *Cryptopsaras couesii* female. This unusual type of mating strategy ensures an endless supply of sperms whenever the female is ready to spawn and represents a major evolutionary advantage in the vast environment of the oceans, where the odds of a male and female encounter is extremely low. 

Like other deep-sea creatures, anglerfishes are subject to a hydrostatic pressure that increases by 1 atm every 10 m of water depth. The high pressure interferes with many cellular processes and can change the rigidity of membranes, alter protein folding and enzyme activity, and interfere with ligand binding to receptors [14]. Many of these effects are prevented in deep-sea animals by the presence of small organic molecules, termed piezolytes [15], which bind tightly to water molecules, prevent water from being forced into the proteins’ interiors, and preserve the overall configuration of the proteins [16]. The deeper an animal lives, the more piezolytes they tend to accumulate in their cells [16,17]. One of the most studied piezolytes in deep-sea animals, the natural osmolyte trimethylamine N-oxide (TMAO), was shown to be capable of increasing the thermodynamic stability of folded proteins and to counteract the hydrostatic pressure-induced protein denaturation [18,19]. The trimethylamine in TMAO is the source of the “fishy” odor of marine animals, and although it is found in shallow water creatures, it is much more abundant in deep-water species, which have a much stronger smell [20]. 

## 2. The Immunological Enigma in Anglerfishes

The immune system of teleost fishes consists of many of the fundamental cell types and operates by utilizing many of the effector mechanisms that are common to all groups of vertebrates. The permanent male-to-female attachment of anglerfish and the establishment of a shared circulatory system represent a putative immunologically intolerable condition of tissue antigen histoincompatibility. A comparable type of parabiosis in vertebrates evokes an extremely potent immune response against the “foreign” major histocompatibility complex (MHC) antigens on the allotransplanted tissue that results in immune rejection of the allograft. From immunological perspectives, the joint circulation of the male and female anglerfish is analogous to that of an intravenously administered allogeneic bone marrow, which, in humans, apart from the combination of monozygotic tweens, results in a concomitant host-versus-graft (HVG) and graft-versus-host (GVH) immune responses. Downregulation of such acute responses in humans is dependent on a cross-matching of donor and recipient MHC haplotypes plus lifelong administration of immunosuppressive drugs that dampens the inflammatory responses against the allograft major and minor histocompatibility antigens [21,22]. 

Much of the enigma of the long-lasting parabiosis in anglerfishes in the absence of even traces of a reciprocal immune rejection was recently solved by Swann et al., who performed a comparative genome analysis of four groups of anglerfish species that differ in their mating strategies [23]. The four groups included anglerfishes that mate through non-attachment (three species), temporal male-to-female attachment (four species), permanent attachment of a single male and a female (three species), and permanent attachment of multiple males to a single female (three species). 

## 3. Defective Expression of MHC Genes in Anglerfishes

Adaptive cell-mediated immunity plays a very significant role in allograft immune-rejection responses where T cells are primed by and respond to peptide antigens that are present on the surface of antigen-presenting cells (APCs). Focusing on T-cell immunity, Swann et al. first analyzed genes of the major histocompatibility complex class I (MHC-I) and class II (MHC-II) receptors that present antigens for immune recognition by CD8+ cytotoxic T (Tc) cells and CD4+ helper T (Th) cells, respectively [24].

MHC-I are polymorphic receptors consisting of a β_2_-microglobulin (β_2_ m) in association with a variable type I membrane-anchored MHC-I heavy chain and are expressed on the outer surface of all nucleated cells in vertebrates [25]. They display a wide variety of peptide fragments, generated from the degradation of ubiquitinated cytosolic proteins in the proteasome. The main physiological function of MHC-I is to enable cells infected with intracellular pathogens, predominantly viruses, to signal the presence of the invader to the immune system and evoke a Tc-cell response against the MHC-I-presented foreign antigen, which eventually leads to elimination of the infected cells [26]. MHC-I molecules also serve as ligands for inhibitory receptors on natural killer (NK) cells, thereby preventing NK cell responses against healthy cells. Furthermore, MHC-I on the cell surface of allotransplants serve as the prime target of Tc-cell responses that lead to acute graft rejection [27]. 

The second class of MHC receptors, the MHC-II, includes a distinct set of dimeric receptors that consist of two types of glycoproteins, termed α and β chains [28]. Both α and β chains are highly polymorphic and are expressed in a constitutive manner on the surface of professional antigen-presenting cells including dendritic cells, mononuclear phagocytes, and B lymphocytes. Their expression is also inducible on the membrane of infected cells by signals provided by interferon γ (IFNγ) [29]. In contrast to MHC-I, MHC-II present peptide antigens derived from extracellular proteins. The extracellular proteins are engulfed by phagocytic cells and undergo lysosomal digestion. The resulting peptides are then loaded into newly synthesized MHC-II molecules which are transported to the cell membrane to present their cargo to Th cells [30]. 

Teleosts are known to display a wide variety of MHC-I molecules, among which *mhc1u* (in which the “*u*” letter represents the Latin word *uno*) [31], and *mhc1z*, which represents an independent lineage that includes both typical and atypical MHC-I molecules [32]. Initial analysis of *mhc* genes in a pair of anglerfish from the *Ceratias holboelli* and *Ceratias uranoscopus* species, which mate via permanent attachment, revealed that each of the male and female partners expresses some unique *mhc* alleles, indicating that in these fish, the reciprocal tolerance to the partner’s alloantigens is not due to genetic identity in *mhc* genes.

Genome analysis of additional anglerfish species revealed that all non-attaching and the majority of the temporarily attaching species possess a relatively large number of U-type MHC-I genes (*mhc1u*) and a few Z-type MHC-I genes (*mhc1z*) [23], in agreement with observations that were made in other teleost species such as Atlantic salmon, zebrafish, cod, and medaka [33]. The anglerfish species tested also possessed a large number of *mhc2a* alleles, indicating that *mhc* gene diversity in temporarily attaching anglerfish species does not represent an immunological barrier in the mating process. 

By contrast, substantial quantitative and qualitative changes in MHC genes were observed in the six species of anglerfish that practice permanent mono-attachment. These fish exhibited a large number of *mhc1z* genes, an abnormal diversity of *mhc2* genes, and an extremely low number and narrow repertoire of *mhc1u* genes that varied between 0 and 2 genes per species. Furthermore, anglerfish species that practice a consortial mating strategy (in which multiple males permanently attach to a single female) were characterized by a complete loss of functional *mhc1u* genes and a near-complete loss of functional genes in other *mhc1* loci.

While genetic alterations in *mhc* genes in anglerfishes may provide a partial explanation of their ability to form a long-lasting permanent attachment, it should be noted that other fish species that mate through non-attachment, including the pipefish (*Syngnathus typhle* [34]), the Atlantic cod (*Gadus morhua* [35]), and the anglerfish (*Lophius piscatorius* [36]) were reported to lack major components of the classical MHC-II pathway. It should be noted that these fishes possess a normal or above normal number of functional MHC-I genes. 

## 4. Antigen Receptors and Coreceptors in T Lymphocytes and Cytotoxic Activity

An immune rejection of allografts in vertebrates is mediated predominantly by two types of effector cells, the B and T lymphocytes, which are equipped with highly-specific invariant B-cell antigen receptors (BCRs) [37] and T-cell antigen receptors (TCRs) [38], respectively. These receptors detect the grafts’ alloantigens and provide signals leading to activation and differentiation of the lymphocytes. Additional accessory cell surface receptors increase the binding affinity of T cells to their targets and provide costimulatory signals essential for the conversion of the lymphocytes into functional effector cells [39]. While MHC-restricted T cells are capable of mediating direct contact with allograft cells and destroying them [40], effector B cells contribute to the immune rejection response indirectly by producing a large number of antibodies. These antibodies bind to the transplant alloantigens and mediate cell destruction by the complement system and by Fc-receptor-positive effector cells such as macrophages and NK cells [41]. 

### 4.1. TCR and TCR-Coupled Signaling Machinery

Both Tc and Th cells express TCR which endows the cells with the ability to specifically recognize foreign or “non-self” antigens and respond against them. The mammalian TCR is composed of an antigen recognition module that includes either TCRα–TCRβ or TCRγ–TCRδ disulfide-linked heterodimers, which associate with a signal transducing module (CD3) that includes six polypeptides: the invariant CD3γ–CD3δ and CD3γ–CD3ε heterodimers and the CD3ζ–CD3ζ homodimer [42]. All six CD3 proteins possess a highly conserved region in their cytoplasmic domain, termed immunoreceptor tyrosine-based activation motif (ITAM), which is essential for signal transduction downstream of peptide antigen-engaged TCR [43].

The fish TCRγδ-encoding ortholog is a single precursor gene, termed *cd3gd*, which gives rise to a polymorphic heterodimeric receptor [44]. The *cd3gd* gene was found to be intact in ceratioids that mate by non-attachment or temporal attachment. In contrast, *cd3gd* could not be detected in anglerfishes that mate by permanent attachment. Furthermore, the genes for the TCR-associated CD3 invariant chains in the latter species were predicted to encode imperfect proteins with non-functional ITAMs, suggesting a defect in these fish species in both the recognition and the signaling modules downstream of the TCRγδ. 

Technical considerations made the analysis of the anglerfish *tcrα* and *tcrβ* genes more difficult to assess due to the high degree of similarity between the *tcrα*/*tcrβ* genes and other immunoglobulin domain-encoding genes. However, sequencing of the *tcrα* and *tcrβ* constant region genes suggested that functional TCRαβ receptors exist in most anglerfish species, except for the species that exhibit permanent attachment of multiple males to a single female.

### 4.2. CD8 Coreceptors

To analyze the status of the TCR costimulatory receptors on the outer membrane of anglerfish T lymphocytes, Swan et al. [23] initially focused on the *cd8α* and *cd8β* genes in Tc lymphocytes that serve as key mediators of allograft rejection. The main function of the invariant heterodimeric CD8αβ surface receptors is to interact with MHC-I presented antigens [45] and thereby regulate Tc cell proliferation, development, activation, and cytotoxic activity [46,47]. Swann et al. have found that functional *cd8α* and *cd8β* genes were missing in all six species tested that mate through permanent attachment, suggesting that these fish are devoid of the classical CD8-regulated T-cell cytotoxic activity. 

### 4.3. CD4 Coreceptors

The MHC-II-restricted CD4+ T cells complement the activity of CD8+ T cells and play an array of critical roles within the adaptive immune system [48]. Through the release of multiple types of cytokines and chemokines they can promote the recruitment of immunocytes to sites of inflammation and regulate their immune activities. In addition, they are essential for the B-cell antibody class switch and affinity maturation processes leading to the formation of antigen-specific high-affinity antibodies [49]. Since CD4+ T-cell-dependent mechanisms also contribute to the allograft rejection response [46], Swann et al. [23] searched for the presence of the two CD4-encoding genes in the different ceratioid species. Their findings indicated that the majority of anglerfish species that practice permanent attachment are devoid of functional CD4 genes. Furthermore, the gene for the CD74 invariant chain, which facilitates MHC-II transport to the endosome and is essential for antigen presentation by MHC-II, was either missing or greatly modified in these fish species. It is therefore suggested that CD4+ T-helper cells are either non-functional or play only a very limited role in the adaptive immunity of anglerfish species that practice permanent attachment. 

### 4.4. Lymphocyte-Mediated Cytotoxicity

The perforin molecule is a key component of the lytic machinery of lymphocytes and is found in the granules of both Tc and NK cells [50,51]. Upon Tc or NK cell activation, perforin molecules are released from the cytolytic granules, insert themselves into the plasma membrane of the target cells, and form pores through which serine proteases insert themselves into the target cells and promote their death [52]. Presence of a functional perforin gene in anglerfishes that mate through permanent attachment suggested that these fish are capable of mediating innate cytotoxic activity, either by cells of the innate immune system, or perhaps by some other unknown cell types. It should be noted that mouse NK cells are capable of mediating an acute immune rejection of allogeneic bone marrow cells [53], suggesting that anglerfish NK cells have also undergone genetic alterations that prevent them from responding against blood cells of the conjoined partner. 

## 5. B-Cell Antigen Receptor and Antibody Production

B lymphocytes are the major cell type in the second arm of the adaptive immune system and the antibodies (immunoglobulins; Igs) they produce are the main component of the humoral immune system. 

While membrane-bound Igs on the surface of B cells serve as antigen-specific receptors that regulate the differentiation and activation of B cells, soluble Igs can physically interact with numerous types of antigens and either neutralize them (i.e., toxins), leading to their endocytosis or phagocytosis (i.e., bacteria) or promote the lysis of the antigen-expressing cells, (i.e., virally infected cells) by the complement system or cytotoxic cells. Furthermore, by interacting with tissue alloantigens, they contribute to the allograft rejection response [46,54,55]. Based on the above information, it was speculated that evolutionary constrains have also altered components or mechanisms of the anglerfish humoral immune system, thereby preventing them from responding against a mate tissue alloantigens by antibody-mediated cytotoxic activity. 

To analyze the anglerfish humoral immune system, Swann et al. searched for the status of the B-cell-specific genes *cd79α* (*Igα*) and *cd79β* (*Igβ*), which encode the B-cell antigen receptor (BCR) subunits, and *igmh*, which encodes the IgM heavy chain [56]. Their studies revealed that the majority of anglerfish species possess the three types of genes, suggesting that their B cells are capable of recognition of foreign antigens and, potentially, respond by antibody production. However, some anglerfish species that practice consortial mating, such as *Photocorynus spiniceps*, acquired deleterious mutations in the *cd79α* and *cd79β* genes, suggesting that they are unable to express a functional BCR and respond by a classical antibody-mediated humoral immunity.

## 6. Generation of Diversity in Lymphocyte Receptors

The formation of the enormous repertoire of antigen receptors in T and B lymphocytes is made possible by the activity of three major types of enzymes that coordinate the rearrangement of three sets of gene segments, namely, variable (V), diversity (D), and joining (J), to form functional variable regions that are then recombined to a constant region. While the recombination-activating gene (RAG)-1 and RAG-2 enzymes are in charge of the gene recombination [57], the terminal deoxynucleotidyl transferase (TdT), a DNA polymerase that catalyzes the stepwise addition of random nucleotides (N) to the V (D)J coding junctions, increases the diversity of the variable regions.

The majority of the anglerfish species tested were found to possess intact *rag1* and *rag2* genes that potentially encode functional enzymes. However, deleterious insertion and deletion mutations in both the *rag1* and *rag2* genes were observed in the genomes of anglerfish species that mediated permanent consortial attachment (*Haplophryne mollis* and *Photocorynus spiniceps*). Based on observations made in mouse and human *rag*-deficient individuals [58,59], these species are likely to be incapable of rearranging functional variable regions in T and B lymphocyte antigen receptors, suggesting that they are unable to mount effective B- or T-cell-mediated immune responses. 

## 7. Affinity Maturation of Immunoglobulins 

The efficiency of the humoral immune response relies on the B cells’ ability to undergo expansion, somatic hypermutation, and affinity maturation, which increase the antibody repertoire and promote the selection of high-affinity antigen-specific antibodies [60,61]. Somatic hypermutations occur predominantly in the variable regions of the Ig heavy and light chain by the activation-induced cytidine deaminase (AICDA) enzyme, which functions as a master regulator of antibody diversification [62,63]. Furthermore, the *aicda* gene contributes to the Ig class switch that is essential for the antibody-mediated allograft rejection response [64,65]. Anglerfish genome analysis confirmed the presence of a functional *aicda* gene in each of the three species that do not form physical attachment during mating. By contrast, the *aicda* gene was undetectable in the genome of ten different anglerfish species that practice either temporary or permanent attachment. 

The results suggest that antibodies of anglerfishes that mate by attachment have lost the ability to undergo affinity maturation and implies also that genetic alterations that enabled mating by attachment were accompanied by a partial or a complete loss of the ability to mount an effective antibody-mediated response. 

## 8. Conclusions

Mammalian fetuses can be considered as semi-allogenic transplants that are tolerated by the maternal’s immune system, which relates to the fetus as “temporary self”. One major mechanism that protects the fetus from rejection by the mother’s immune system is mediated by the placenta, which functions as an immunological barrier between the fetus and the mother and creates an immunologically privileged site for the fetus. Multiple additional mechanisms prevent maternal immune responses towards the fetus while maintaining the capacity to mount a defense against infectious organisms. These include the lack or altered expression of certain alloantigens on fetal tissues, the induction of maternal tolerance against the fetal tissue, and site-specific suppression of maternal immune responses at the fetal–maternal interface [66].

None of the above mechanisms can explain the immune tolerance of an anglerfish female to its male counterpart which attaches itself to her skin, the most potent immune organ in the body which is always in contact with a large number of antigens and pathogens. 

To learn about the anglerfish immune system, a comparative genomic analysis was performed in anglerfish species that differ in their mating strategies. The results demonstrated major differences between species in genes encoding proteins that are critical for the execution of adaptive immune responses. The main findings implied that species that fuse permanently during mating lack critical components of the immune system and are therefore incapable of rejecting the co-joined allogeneic partner. It is speculated that adaptation of anglerfishes to their unique environment resulted in multiple anatomical, physiological, and behavioral changes that led to distinctive mating strategies and that these alterations coevolved with genetic changes that led to a partial or complete loss of genes that regulate certain aspects of the immune response against the allogeneic parabiosed partner.

Studies of vertebrates, such as mouse and human, indicated that a combined loss of the two major arms of the adaptive immune system results in severe combined immunodeficiency (SCID), a fatal condition due to the severe malfunction of the immune system [67]. It is currently unclear how anglerfishes manage to defend themselves from pathogens in the absence of a functional adaptive immune system. It is speculated that loss of components of the adaptive immunity is compensated by a gain of function of some unknown alternative adaptive and/or innate immune system mechanisms that provide a better protection from pathogens without compromising the immune tolerance to the conjoined parasitic male.
